# Supplementary material for: Habitual Diet Pattern Associations with Gut Microbiome Diversity and Composition: Results from a Chinese Adult Cohort
Source: Nutrients. 2022 Jun 25;14(13):2639. doi: 10.3390/nu14132639 (PMC9268000; doi:10.3390/nu14132639)
Supplement: Supplementary file 1 [file nutrients-14-02639-s001.zip › nutrients-1749546-supplementary.pdf]

Supplementary materials

Supplementary Method 1. Dietary pattern analysis

The pattern loadings from the factor analysis in Supplementary Table S1 showed that the first factor had high loadings on eggs, dairy, whole grain and fruits (absolute value of factor loading larger than 0.45). The second factor showed high loadings on refined grains and fruits and a negative high loading on cooked meat. The third factor has high loadings on red meat and white meat. These three factors totally explained 50% of the whole variance of food intake frequencies. We subsequently conducted a cluster analysis based on the factor scores for each factor and each participant calculated by summing the consumption of each food group that was weighted by its factor loading. The optimal number of clusters was selected by 30 indices serving as measurement metrics of clustering performance using R package "NbClust". Using K-means clustering method, we finally cluster the participants into 3 groups according the aforementioned common factor scores.

Supplementary Method 2. MaAsLin

MaAsLin is a multivariate statistical framework that finds associations between metadata and relative abundances of specific microbial community members. MaAsLin first deconfounds and selects, among metadata, factors most potentially associated with microbial abundance. Selected factors are then included in general linear models using metadata as predictors and taxa arcsin-square root-transformed abundances as the response. Phyla, genera, and species were filtered out if their relative abundance and prevalence were <0.01% and <1%, respectively. The number of tests performed was high for MaAsLin, therefore P values were adjusted for multiple testing using the Benjamini–Hochberg method with a 10% false discovery rate.

Supplementary Table S1. Factor loading matrix of major factors by principal component analysis with varimax rotation.

	PA1	PA2	PA3
Red meat	-0.051	0.149	**0.678**
White meat	0.142	0.007	**0.644**
Eggs	**0.678**	-0.045	0.172
Dairy	**0.651**	-0.419	0.159
Cooked meat	0.3	**-0.498**	0.322
Refined grain	-0.047	**0.707**	0.17
Whole grain	**0.65**	-0.043	-0.003
Fruits	**0.689**	0.065	-0.042
Vegetables	0.056	**0.756**	0.122
Explained variance	21%	17%	12%

Figures in bold indicate absolute factor loading are more than 0.45. PA, Principal axis.

Supplementary Table S2. Classification of subjects by cluster analysis using factor score.

	Cluster A	Cluster B	Cluster C
Factor 1	-0.413 ± 0.438	1.708 ± 0.721	0.131 ± 0.653
Factor 2	0.457 ± 0.333	0.351 ± 0.598	-1.253 ± 0.611
Factor 3	0.077 ± 0.717	0.173 ± 1.139	-0.267 ± 0.763

Supplementary Table S3. Dietary patterns identified by K-means clustering.

	Cluster A	Cluster B	Cluster C
Red meat	7.547 ± 4.123	7.011 ± 4.744	5.077 ± 3.066
White meat	4.579 ± 3.68	5.756 ± 4.572	4.132 ± 2.718
Eggs	3.621 ± 2.782	9.856 ± 3.916	5.143 ± 2.459
Dairy	0.726 ± 1.64	6.367 ± 4.536	4.659 ± 3.089
Cooked meat	0.116 ± 0.52	2.211 ± 4.312	2.407 ± 3.565
Refined grain	13.679 ± 1.699	13.356 ± 2.27	6.385 ± 3.66
Whole grain	2.186 ± 2.546	10.056 ± 4.665	4.566 ± 2.968
Fruits	3.749 ± 3.619	12.233 ± 3.435	5.154 ± 2.761
Vegetables	13.133 ± 2.354	13.533 ± 1.756	6.363 ± 3.262

Supplementary Table S4. Region distributions of 702 participants according to the established dietary patterns.

Region	Cluster A	Cluster B	Cluster C
Changsha, Hunan province	69 (36.3%)	32 (16.8%)	89 (46.8%)
Hefei, Anhui province	47 (51.1%)	11 (12%)	34 (37%)
Kunming, Yunan province	7 (50.0%)	5 (35.7%)	2 (14.3%)
Lanxi, Zhejiang province	137 (89%)	1 (0.6%)	16 (10.4%)
Taizhou, Zhejiang province	157 (95.7%)	4 (2.4%)	3 (1.8%)
Xuzhou, Jiangsu province	13 (14.8%)	37 (42%)	38 (43.2%)

Supplementary Table S5. Sensitivity analysis of associations between food intakes and gut microbial profiles using MaAsLins with population at a high risk of intestinal diseases removed, *N*=479.

Food group	Phylum	Class | Order | Family	Genus	Species	Value	Coef[1]	Coverage (%)[2]	Pval[3]	Qval[4]
Red meat	*Firmicutes*	*Bacilli* | *Lactobacillales* | *Leuconostocaceae*	*Weissella*	*uncultured organism*	pd	-0.0507	27.14%	<0.0001	0.0711
Red meat	*Firmicutes*	*Bacilli* | *Lactobacillales* | *Leuconostocaceae*	*Weissella*		pd	-0.0502	28.81%	<0.0001	0.0711
Red meat	*Firmicutes*	*Bacilli* | *Lactobacillales* | *Leuconostocaceae*	*Weissella*	*uncultured organism*	mul_pw	-0.0471	27.14%	0.0001	0.0804
Cooked meat	*Bacteroidetes*	*Bacteroidia* | *Bacteroidales* | *Barnesiellaceae*	*Coprobacter*		pd	0.0172	14.61%	<0.0001	0.0195
Cooked meat	*Firmicutes*	*Clostridia* | *Clostridiales* | *Lachnospiraceae*	*Eubacterium eligens group*	*uncultured organism*	mul_pw	0.0493	65.14%	<0.0001	0.0430
Cooked meat	*Firmicutes*	*Clostridia* | *Clostridiales* | *Lachnospiraceae*	*uncultured*	*uncultured organism*	mul_pw	0.0123	34.24%	<0.0001	0.0430
Cooked meat	*Firmicutes*	*Clostridia* | *Clostridiales* | *Lachnospiraceae*	*Eubacterium eligens group*		mul_pw	0.0491	65.14%	<0.0001	0.0430
Cooked meat	*Bacteroidetes*	*Bacteroidia* | *Bacteroidales* | *Prevotellaceae*	*Paraprevotella*	*uncultured organism*	pd	0.0299	11.27%	0.0001	0.0495
Whole grain	*Firmicutes*	*Negativicutes* | *Veillonellales* | *Veillonellaceae*	*Megasphaera*	*uncultured organism*	mul_pd	0.0515	13.15%	<0.0001	0.0425
Refined grain	*Firmicutes*	*Bacilli* | *Lactobacillales* | *Lactobacillaceae*		*uncultured organism*	pw	0.0959	12.94%	<0.0001	0.0026

Refined grain	*Firmicutes*	*Clostridia* | *Clostridiales* | *Ruminococcaceae*	*Eubacterium coprostanoligenes group*	*uncultured organism*	mul_pd	-0.0443	18.58%	<0.0001	0.0249
Refined grain	*Firmicutes*	*Clostridia* | *Clostridiales* | *Ruminococcaceae*	*Eubacterium coprostanoligenes group*	*uncultured organism*	pd	-0.0455	18.58%	<0.0001	0.0249
Refined grain	*Firmicutes*	*Bacilli* | *Lactobacillales* | *Lactobacillaceae*	*Lactobacillus*	*Lactobacillus mucosae*	pw	0.0677	15.24%	0.0001	0.0506
Refined grain	*Firmicutes*	*Bacilli* | *Lactobacillales* | *Lactobacillaceae*	*Lactobacillus*		pw	0.1565	49.69%	0.0001	0.0750
Refined grain	*Firmicutes*	*Clostridia* | *Clostridiales* | *Ruminococcaceae*	*Eubacterium coprostanoligenes group*	*uncultured organism*	mul_pw	-0.0408	18.58%	0.0002	0.0842
Refined grain	*Firmicutes*	*Clostridia* | *Clostridiales* | *Ruminococcaceae*	*Ruminococcaceae UCG 013*		pw	-0.0734	74.95%	0.0002	0.0842
Vegetables	*Firmicutes*	*Clostridia* | *Clostridiales* | *Ruminococcaceae*	*Eubacterium coprostanoligenes group*	*uncultured organism*	mul_pd	-0.3279	24.84%	<0.0001	<0.0001
Vegetables	*Firmicutes*	*Clostridia* | *Clostridiales* | *Ruminococcaceae*	*Eubacterium coprostanoligenes group*	*uncultured organism*	pd	-0.3281	24.84%	<0.0001	<0.0001
Vegetables	*Firmicutes*	*Clostridia* | *Clostridiales* | *Ruminococcaceae*	*Eubacterium coprostanoligenes group*	*uncultured organism*	mul_pw	-0.3233	24.84%	<0.0001	<0.0001
Vegetables	*Firmicutes*	*Clostridia* | *Clostridiales* | *Ruminococcaceae*	*Eubacterium coprostanoligenes group*	*uncultured organism*	pw	-0.3290	24.84%	<0.0001	<0.0001
Vegetables	*Firmicutes*	*Clostridia* | *Clostridiales* | *Ruminococcaceae*	*Eubacterium coprostanoligenes group*		pd	-0.2894	70.35%	<0.0001	0.0001
Vegetables	*Firmicutes*	*Clostridia* | *Clostridiales* | *Ruminococcaceae*	*Eubacterium coprostanoligenes group*		mul_pd	-0.2822	70.35%	<0.0001	0.0001
Vegetables	*Firmicutes*	*Clostridia* | *Clostridiales* | *Ruminococcaceae*	*Eubacterium coprostanoligenes group*		mul_pw	-0.2795	70.35%	<0.0001	0.0001

Vegetables	*Firmicutes*	*Erysipelotrichia* | *Erysipelotrichales* | *Erysipelotrichaceae*	*Turicibacter*	*uncultured organism*	pd	-0.0774	13.36%	<0.0001	0.0002
Vegetables	*Firmicutes*	*Erysipelotrichia* | *Erysipelotrichales* | *Erysipelotrichaceae*	*Turicibacter*	*uncultured organism*	mul_pw	-0.0788	13.36%	<0.0001	0.0002
Vegetables	*Firmicutes*	*Erysipelotrichia* | *Erysipelotrichales* | *Erysipelotrichaceae*	*Turicibacter*	*uncultured organism*	pw	-0.0812	13.36%	<0.0001	0.0002
Vegetables	*Firmicutes*	*Erysipelotrichia* | *Erysipelotrichales* | *Erysipelotrichaceae*	*Turicibacter*	*uncultured organism*	mul_pd	-0.0758	13.36%	<0.0001	0.0002
Vegetables	*Firmicutes*	*Clostridia* | *Clostridiales* | *Ruminococcaceae*	*Eubacterium coprostanoligenes group*		pw	-0.2747	70.35%	<0.0001	0.0003
Vegetables	*Bacteroidetes*	*Bacteroidia* | *Bacteroidales* | *Barnesiellaceae*	*Barnesiella*	*uncultured organism*	pd	-0.0533	10.44%	<0.0001	0.0079
Vegetables	*Bacteroidetes*	*Bacteroidia* | *Bacteroidales* | *Barnesiellaceae*	*Barnesiella*	*uncultured organism*	mul_pw	-0.0532	10.44%	<0.0001	0.0097
Vegetables	*Bacteroidetes*	*Bacteroidia* | *Bacteroidales* | *Barnesiellaceae*	*Barnesiella*	*uncultured organism*	mul_pd	-0.0517	10.44%	<0.0001	0.0115
Vegetables	*Firmicutes*	*Clostridia* | *Clostridiales* | *Ruminococcaceae*	*uncultured*	*uncultured organism*	mul_pw	-0.0611	14.82%	0.0001	0.0169
Vegetables	*Firmicutes*	*Clostridia* | *Clostridiales* |	*uncultured*	*uncultured*	pd	-0.0598	14.82%	0.0001	0.0176

		Ruminococcaceae		*organism*					
Vegetables	*Firmicutes*	*Clostridia | Clostridiales | Ruminococcaceae*	*Ruminococcaceae UCG 005*	*uncultured organism*	pw	-0.0600	11.69%	0.0001	0.0221
Vegetables	*Firmicutes*	*Clostridia | Clostridiales | Ruminococcaceae*	*Oscillibacter*	*uncultured organism*	pd	-0.0269	10.02%	0.0001	0.0233
Vegetables	*Firmicutes*	*Clostridia | Clostridiales | Ruminococcaceae*	*Oscillibacter*	*uncultured organism*	pw	-0.0281	10.02%	0.0001	0.0233
Vegetables	*Firmicutes*	*Clostridia | Clostridiales | Ruminococcaceae*	*uncultured*	*uncultured organism*	mul_pd	-0.0578	14.82%	0.0001	0.0233
Vegetables	*Firmicutes*	*Clostridia | Clostridiales | Ruminococcaceae*	*Ruminococcaceae UCG 002*	*uncultured organism*	pw	-0.1215	23.80%	0.0001	0.0241
Vegetables	*Bacteroidetes*	*Bacteroidia | Bacteroidales | Barnesiellaceae*	*Barnesiella*	*uncultured organism*	pw	-0.0500	10.44%	0.0001	0.0242
Vegetables	*Firmicutes*	*Clostridia | Clostridiales | Ruminococcaceae*	*Ruminococcaceae UCG 005*	*uncultured organism*	mul_pw	-0.0563	11.69%	0.0001	0.0242
Vegetables	*Firmicutes*	*Clostridia | Clostridiales | Ruminococcaceae*	*uncultured*	*uncultured organism*	pw	-0.0600	14.82%	0.0001	0.0242
Vegetables	*Firmicutes*	*Clostridia | Clostridiales | Ruminococcaceae*	*Oscillibacter*	*uncultured organism*	mul_pd	-0.0260	10.02%	0.0001	0.0262
Vegetables	*Firmicutes*	*Clostridia | Clostridiales | Ruminococcaceae*	*Oscillibacter*	*uncultured organism*	mul_pw	-0.0260	10.02%	0.0002	0.0312
Vegetables	*Firmicutes*	*Clostridia | Clostridiales | Ruminococcaceae*	*Ruminococcaceae UCG 005*	*uncultured organism*	pd	-0.0540	11.69%	0.0002	0.0312
Vegetables	*Firmicutes*	*Clostridia | Clostridiales | Christensenellaceae*	*Christensenellaceae R 7 group*	*uncultured organism*	pd	-0.1159	28.39%	0.0002	0.0327

Vegetables	*Actinobacteria*	*Coriobacteriia | Coriobacteriales | Eggerthellaceae*	*Adlercreutzia*	*uncultured organism*	pd	-0.0774	45.93%	0.0002	0.0332
Vegetables	*Actinobacteria*	*Coriobacteriia | Coriobacteriales | Eggerthellaceae*	*Adlercreutzia*		pd	-0.0774	45.93%	0.0002	0.0332
Vegetables	*Firmicutes*	*Clostridia | Clostridiales | Christensenellaceae*	*Christensenellaceae R 7 group*	*uncultured organism*	mul_pd	-0.1146	28.39%	0.0002	0.0335
Vegetables	*Actinobacteria*	*Coriobacteriia | Coriobacteriales | Eggerthellaceae*	*Adlercreutzia*	*uncultured organism*	mul_pd	-0.0762	45.93%	0.0003	0.0360
Vegetables	*Firmicutes*	*Clostridia | Clostridiales | Ruminococcaceae*	*Ruminococcaceae UCG 005*	*uncultured organism*	mul_pd	-0.0525	11.69%	0.0003	0.0360
Vegetables	*Actinobacteria*	*Coriobacteriia | Coriobacteriales | Eggerthellaceae*	*Adlercreutzia*		mul_pd	-0.0762	45.93%	0.0003	0.0360
Vegetables	*Firmicutes*	*Erysipelotrichia | Erysipelotrichales | Erysipelotrichaceae*	*Turicibacter*		pw	-0.1032	42.59%	0.0003	0.0407
Vegetables	*Firmicutes*	*Clostridia | Clostridiales | Ruminococcaceae*	*Ruminococcaceae UCG 002*	*uncultured organism*	mul_pd	-0.1067	23.80%	0.0003	0.0421
Vegetables	*Firmicutes*	*Clostridia | Clostridiales | Ruminococcaceae*	*Ruminococcaceae UCG 003*	*uncultured organism*	pw	-0.1349	57.83%	0.0003	0.0435
Vegetables	*Firmicutes*	*Clostridia | Clostridiales | Christensenellaceae*	*Christensenellaceae R 7 group*	*uncultured organism*	pw	-0.1151	28.39%	0.0004	0.0492
Vegetables	*Firmicutes*	*Clostridia | Clostridiales |*	*Ruminococcaceae UCG 002*	*uncultured*	pd	-0.1034	23.80%	0.0005	0.0583

		Ruminococcaceae		*organism*					
Vegetables	*Firmicutes*	*Clostridia | Clostridiales | Ruminococcaceae*	*Ruminococcaceae UCG 003*	*uncultured organism*	mul_pw	-0.1036	23.80%	0.0006	0.0606
Vegetables	*Actinobacteria*	*Coriobacteriia | Coriobacteriales | Eggerthellaceae*	*Adlercreutzia*	*uncultured organism*	mul_pw	-0.0722	45.93%	0.0007	0.0629
Vegetables	*Actinobacteria*	*Coriobacteriia | Coriobacteriales | Eggerthellaceae*	*Adlercreutzia*		mul_pw	-0.0722	45.93%	0.0007	0.0629
Vegetables	*Firmicutes*	*Clostridia | Clostridiales | Ruminococcaceae*	*Ruminococcaceae UCG 002*	*uncultured organism*	pd	-0.1215	57.83%	0.0007	0.0674
Vegetables	*Firmicutes*	*Clostridia | Clostridiales | Christensenellaceae*	*Christensenellaceae R 7 group*	*uncultured organism*	mul_pw	-0.1046	28.39%	0.0009	0.0796
Vegetables	*Firmicutes*	*Erysipelotrichia | Erysipelotrichales | Erysipelotrichaceae*	*Turicibacter*		mul_pw	-0.0913	42.59%	0.0009	0.0802
Vegetables	*Actinobacteria*	*Coriobacteriia | Coriobacteriales | Eggerthellaceae*	*Adlercreutzia*	*uncultured organism*	pw	-0.0722	45.93%	0.0010	0.0835
Vegetables	*Actinobacteria*	*Coriobacteriia | Coriobacteriales | Eggerthellaceae*	*Adlercreutzia*		pw	-0.0722	45.93%	0.0010	0.0835
Vegetables	*Firmicutes*	*Clostridia | Clostridiales | Ruminococcaceae*	*Ruminococcaceae UCG 002*	*uncultured organism*	mul_pd	-0.1159	57.83%	0.0012	0.0950
Vegetables	*Firmicutes*	*Erysipelotrichia | Erysipelotrichales |*	*Turicibacter*		pd	-0.0880	42.59%	0.0012	0.0964

		Erysipelotrichaceae							
Vegetables	*Firmicutes*	*Clostridia* | *Clostridiales* | *Lachnospiraceae*	*uncultured*	*uncultured organism*	pd	-0.0550	10.44%	0.0013	0.0998

[1]For categorical features in MaAsLins analysis, the specific feature level for which the coefficient and significance of association is being reported.

[2]Prevalence of bacterial taxa in the study sample, equals to the total of number of samples in which the feature is non-zero divided by the total number of samples used in the model.

[3]*P* value for MaAsLin adjusted for age, sex, BMI, smoking status, alcohol consumption and physical activity; computed using the Maaslin2 package on R.

[4]Corrected *P* value by the Benjamini–Hochberg method (10% false discovery rate).

Supplementary Table S6. Sensitivity analysis of associations between food intakes and gut microbial profiles using MaAsLins among population from a single province, *N*=318.

Food group	Phylum	Class | Order | Family	Genus	Species	Value	Coef[1]	Coverage (%)[2]	Pval[3]	Qval[4]
Red meat	*Firmicutes*	*Bacilli | Lactobacillales | Leuconostocaceae*	*Weissella*	*uncultured organism*	pd	-0.0660	33.02%	<0.0001	0.0910
Red meat	*Firmicutes*	*Bacilli | Lactobacillales | Leuconostocaceae*	*Weissella*		pd	-0.0661	33.96%	<0.0001	0.0910
Cooked meat	*Firmicutes*	*Clostridia | Clostridiales | Lachnospiraceae*	*Eubacterium xylanophilum group*	*uncultured organism*	mul_pw	0.0628	12.58%	<0.0001	0.0015
Cooked meat	*Firmicutes*	*Clostridia | Clostridiales | Lachnospiraceae*	*Eubacterium xylanophilum group*		mul_pw	0.0628	12.58%	<0.0001	0.0015
Cooked meat	*Synergistetes*	*Synergistia | Synergistales | Synergistaceae*	*Pyramidobacter*	*Pyramidobacter piscolens*	mul_pw	0.0437	13.84%	<0.0001	0.0166
Cooked meat	*Synergistetes*	*Synergistia | Synergistales | Synergistaceae*	*Pyramidobacter*		mul_pw	0.0429	15.41%	<0.0001	0.0291
Cooked meat	*Firmicutes*	*Clostridia | Clostridiales | Ruminococcaceae*	*Ruminococcus 1*	*uncultured organism*	mul_pw	0.1148	22.33%	0.0001	0.0941
Whole grain	*Firmicutes*	*Bacilli | Lactobacillales | Leuconostocaceae*	*Leuconostoc*	*uncultured organism*	mul_pw	-0.0086	23.90%	<0.0001	0.0134
Whole grain	*Firmicutes*	*Bacilli | Lactobacillales | Leuconostocaceae*	*Leuconostoc*		mul_pw	-0.0086	23.90%	<0.0001	0.0134
Refined grain	*Firmicutes*	*Bacilli | Lactobacillales | Lactobacillaceae*	*Lactobacillus*	*uncultured organism*	pw	0.1901	14.78%	<0.0001	<0.0001
Refined grain	*Firmicutes*	*Bacilli | Lactobacillales | Lactobacillaceae*	*Lactobacillus*	*Lactobacillus mucosae*	pw	0.1333	21.70%	<0.0001	0.0072
Vegetables	*Firmicutes*	*Bacilli | Lactobacillales |*	*Lactobacillus*	*uncultured*	pw	0.2231	14.78%	<0.0001	<0.0001

		Lactobacillaceae		*organism*					
Vegetables	*Firmicutes*	*Bacilli* | *Lactobacillales* | *Lactobacillaceae*	*Lactobacillus*	*Lactobacillus mucosae*	pw	0.1412	21.70%	<0.0001	<0.0001
Vegetables	*Firmicutes*	*Bacilli* | *Lactobacillales* | *Lactobacillaceae*	*Lactobacillus*		pw	0.2730	53.46%	<0.0001	<0.0001
Vegetables	*Firmicutes*	*Clostridia* | *Clostridiales* | *Ruminococcaceae*	*Ruminiclostridium 5*		pd	0.0118	59.43%	<0.0001	0.0268
Cluster	*Actinobacteria*	*Actinobacteria* | *Bifidobacteriales* | *Bifidobacteriaceae*	*Bifidobacterium*		C	0.0661	77.04%	<0.0001	0.0122

[1]For categorical features in MaAsLins analysis, the specific feature level for which the coefficient and significance of association is being reported.

[2]Prevalence of bacterial taxa in the study sample, equals to the total of number of samples in which the feature is non-zero divided by the total number of samples used in the model.

[3]*P* value for MaAsLin adjusted for age, sex, BMI, smoking status, alcohol consumption and physical activity; computed using the Maaslin2 package on R.

[4]Corrected *P* value by the Benjamini–Hochberg method (10% false discovery rate).

Supplementary Table S7. Sensitivity analysis of associations between food intakes and gut microbial profiles using MaAsLins among population with normal BMI values, *N*=342.

Food group	Phylum	Class | Order | Family	Genus	Species	Value	Coef[1]	Coverage (%)[2]	Pval[3]	Qval[4]
Whole grain	*Firmicutes*	Negativicutes | Veillonellales | Veillonellaceae	Megasphaera	uncultured organism	mul_pd	0.0639	13.45%	<0.0001	0.0016
Whole grain	*Firmicutes*	Negativicutes | Veillonellales | Veillonellaceae	Megasphaera		mul_pd	0.0600	24.56%	<0.0001	0.0082
Refined grain	*Firmicutes*	Clostridia | Clostridiales | Ruminococcaceae	Eubacterium coprostanoligenes group	uncultured organism	pd	-0.0428	15.50%	<0.0001	0.0218
Refined grain	*Firmicutes*	Clostridia | Clostridiales | Ruminococcaceae	Eubacterium coprostanoligenes group	uncultured organism	mul_pd	-0.0404	15.50%	0.0001	0.0233
Vegetables	*Actinobacteria*	*Coriobacteriia* | *Coriobacteriales* | *Coriobacteriaceae*	*Collinsella*	*uncultured organism*	mul_pd	-0.1520	12.57%	<0.0001	<0.0001
Vegetables	*Actinobacteria*	*Coriobacteriia* | *Coriobacteriales* | *Coriobacteriaceae*	*Collinsella*	*uncultured organism*	pd	-0.1547	12.57%	<0.0001	<0.0001
Vegetables	*Actinobacteria*	*Coriobacteriia* | *Coriobacteriales* | *Coriobacteriaceae*	*Collinsella*	*uncultured organism*	mul_pw	-0.1595	12.57%	<0.0001	<0.0001
Vegetables	*Actinobacteria*	*Coriobacteriia* | *Coriobacteriales* | *Coriobacteriaceae*	*Collinsella*	*uncultured organism*	pw	-0.1585	12.57%	<0.0001	0.0001
Vegetables	*Firmicutes*	*Bacilli* | *Lactobacillales* | *Leuconostocaceae*	*Leuconostoc*	*uncultured organism*	mul_pd	-0.0403	19.30%	<0.0001	0.0043
Vegetables	*Firmicutes*	*Bacilli* | *Lactobacillales* | *Leuconostocaceae*	*Leuconostoc*	*uncultured organism*	pd	-0.0409	19.30%	<0.0001	0.0043
Vegetables	*Firmicutes*	*Bacilli* | *Lactobacillales* |	*Leuconostoc*		mul_pd	-0.0403	19.30%	<0.0001	0.0043

		Leuconostocaceae							
Vegetables	*Firmicutes*	*Bacilli* | *Lactobacillales* | *Leuconostocaceae*	*Leuconostoc*		pd	-0.0409	19.30%	<0.0001	0.0043
Vegetables	*Firmicutes*	*Bacilli* | *Lactobacillales* | *Leuconostocaceae*	*Leuconostoc*	*uncultured organism*	mul_pw	-0.0415	19.30%	<0.0001	0.0063
Vegetables	*Firmicutes*	*Clostridia* | *Clostridiales* | *Ruminococcaceae*	*Eubacterium coprostanoligenes group*	*uncultured organism*	pd	-0.0993	23.68%	<0.0001	0.0063
Vegetables	*Firmicutes*	*Bacilli* | *Lactobacillales* | *Leuconostocaceae*	*Leuconostoc*		mul_pw	-0.0415	19.30%	<0.0001	0.0063
Vegetables	*Firmicutes*	*Clostridia* | *Clostridiales* | *Ruminococcaceae*	*Eubacterium coprostanoligenes group*	*uncultured organism*	mul_pd	-0.0956	23.68%	<0.0001	0.0092
Vegetables	*Firmicutes*	*Bacilli* | *Lactobacillales* | *Leuconostocaceae*	*Leuconostoc*	*uncultured organism*	pw	-0.0429	19.30%	0.0001	0.0112
Vegetables	*Firmicutes*	*Bacilli* | *Lactobacillales* | *Leuconostocaceae*	*Leuconostoc*		pw	-0.0429	19.30%	0.0001	0.0112
Vegetables	*Firmicutes*	*Clostridia* | *Clostridiales* | *Ruminococcaceae*	*Eubacterium coprostanoligenes group*	*uncultured organism*	pw	-0.1076	23.68%	0.0001	0.0120
Vegetables	*Firmicutes*	*Clostridia* | *Clostridiales* | *Ruminococcaceae*	*Eubacterium coprostanoligenes group*	*uncultured organism*	mul_pw	-0.0900	23.68%	0.0003	0.0519
Vegetables	*Firmicutes*	*Clostridia* | *Clostridiales* | *Lachnospiraceae*	*Eubacterium ventriosum group*	*uncultured organism*	pw	0.0609	32.46%	0.0005	0.0698
Vegetables	*Firmicutes*	*Clostridia* | *Clostridiales* |	*Eubacterium*		pw	0.0688	62.87%	0.0007	0.0929

		Lachnospiraceae	*ventriosum group*						
Cluster	*Actinobacteria*	*Actinobacteria* | *Bifidobacteriales* | *Bifidobacteriaceae*	*Bifidobacterium*		B	0.0651	78.36%	<0.0001	0.0057
Cluster	*Firmicutes*	*Negativicutes* | *Veillonellales* | *Veillonellaceae*	*Megasphaera*	*uncultured organism*	B	0.0421	13.45%	0.0004	0.0817
Cluster	*Actinobacteria*	*Actinobacteria* | *Bifidobacteriales* | *Bifidobacteriaceae*	*Bifidobacterium*	*uncultured organism*	B	0.0430	43.27%	0.0005	0.0851

[1]For categorical features in MaAsLins analysis, the specific feature level for which the coefficient and significance of association is being reported.

[2]Prevalence of bacterial taxa in the study sample, equals to the total of number of samples in which the feature is non-zero divided by the total number of samples used in the model.

[3]*P* value for MaAsLin adjusted for age, sex, BMI, smoking status, alcohol consumption and physical activity; computed using the Maaslin2 package on R.

[4]Corrected *P* value by the Benjamini–Hochberg method (10% false discovery rate).

Supplementary Table S8. Contingency correlation coefficient between the region variable and dietary variables

**Food group**	**Cramer's V**	**Pval**
Red meat	0.246	<0.0001
White meat	0.227	<0.0001
Eggs	0.279	<0.0001
Dairy	0.332	<0.0001
Cooked meat	0.256	<0.0001
Refined grain	0.252	<0.0001
Whole grain	0.368	<0.0001
Fruits	0.287	<0.0001
Vegetables	0.251	<0.0001
Cluster	0.462	<0.0001

Supplementary figure legends

Supplementary Figure S1. Workflow diagram for the subject enrollment and exclusion.

Supplementary Figure S2. Parallel analysis scree plot to determine the number of factors for factor analysis.

Supplementary Figure S3. α-diversity indexes (richness, chao 1, simpson index, pielou index, ACE and faith-pd index in different intake frequency groups of red meat.

Supplementary Figure S4. α-diversity indexes (richness, chao 1, simpson index, pielou index, ACE and faith-pd index in different intake frequency groups of white meat.

Supplementary Figure S5. α-diversity indexes (richness, chao 1, simpson index, pielou index, ACE and faith-pd index in different intake frequency groups of cooked meat.

Supplementary Figure S6. α-diversity indexes (richness, chao 1, simpson index, pielou index, ACE and faith-pd index in different intake frequency groups of eggs.

Supplementary Figure S7. α-diversity indexes (richness, chao 1, simpson index, pielou index, ACE and faith-pd index in different intake frequency groups of dairy products.

Supplementary Figure S8. α-diversity indexes (richness, chao 1, simpson index, pielou index, ACE and faith-pd index in different intake frequency groups of refined grain.

Supplementary Figure S9. α-diversity indexes (richness, chao 1, simpson index, pielou index, ACE and faith-pd index in different intake frequency groups of whole grain.

Supplementary Figure S10. α-diversity indexes (richness, chao 1, simpson index, pielou index, ACE and faith-pd index in different intake frequency groups of fruits.

Supplementary Figure S11. α-diversity indexes (richness, chao 1, simpson index, pielou index, ACE and faith-pd index in different intake frequency groups of vegetables.

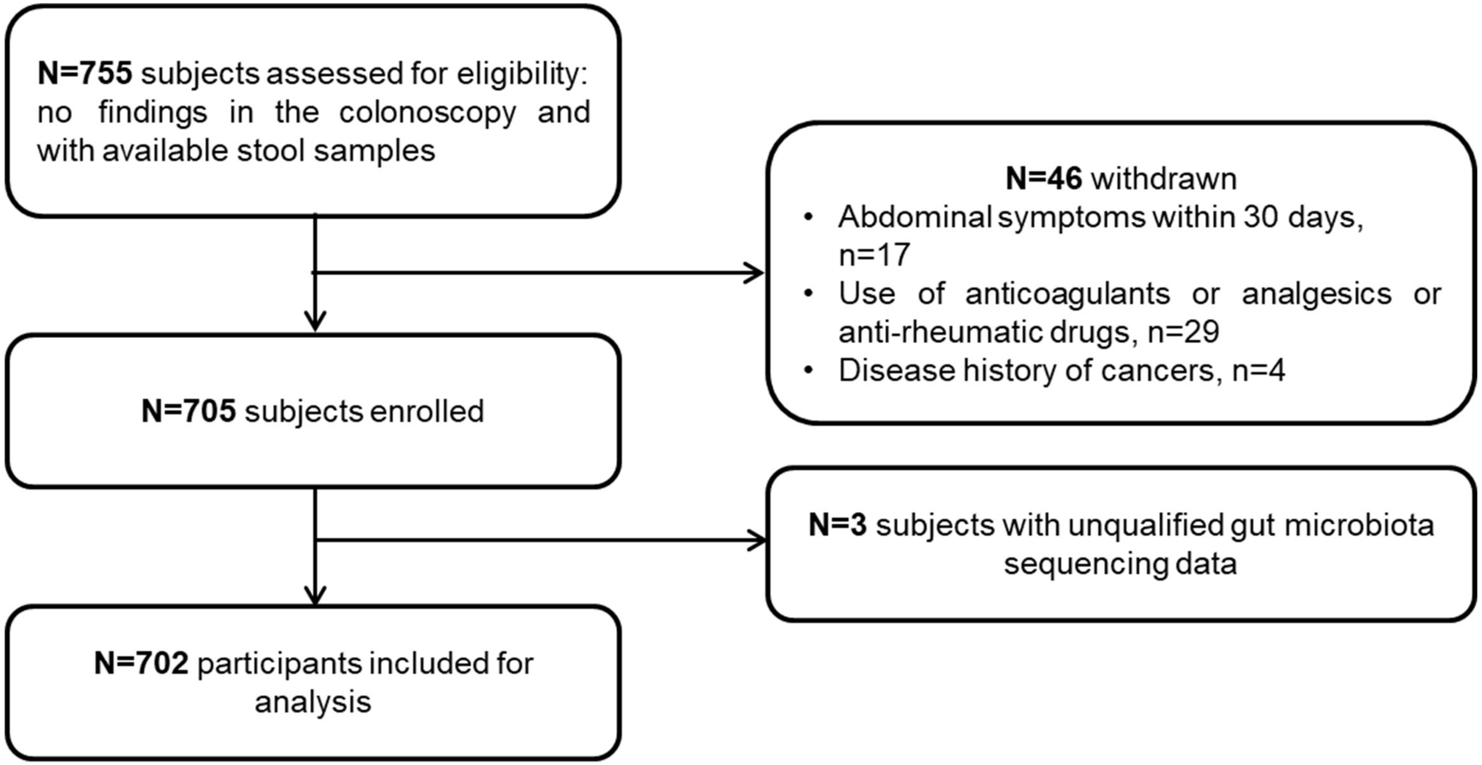

Supplementary Figure S1. Workflow diagram for the subjects enrollment and exclusion.

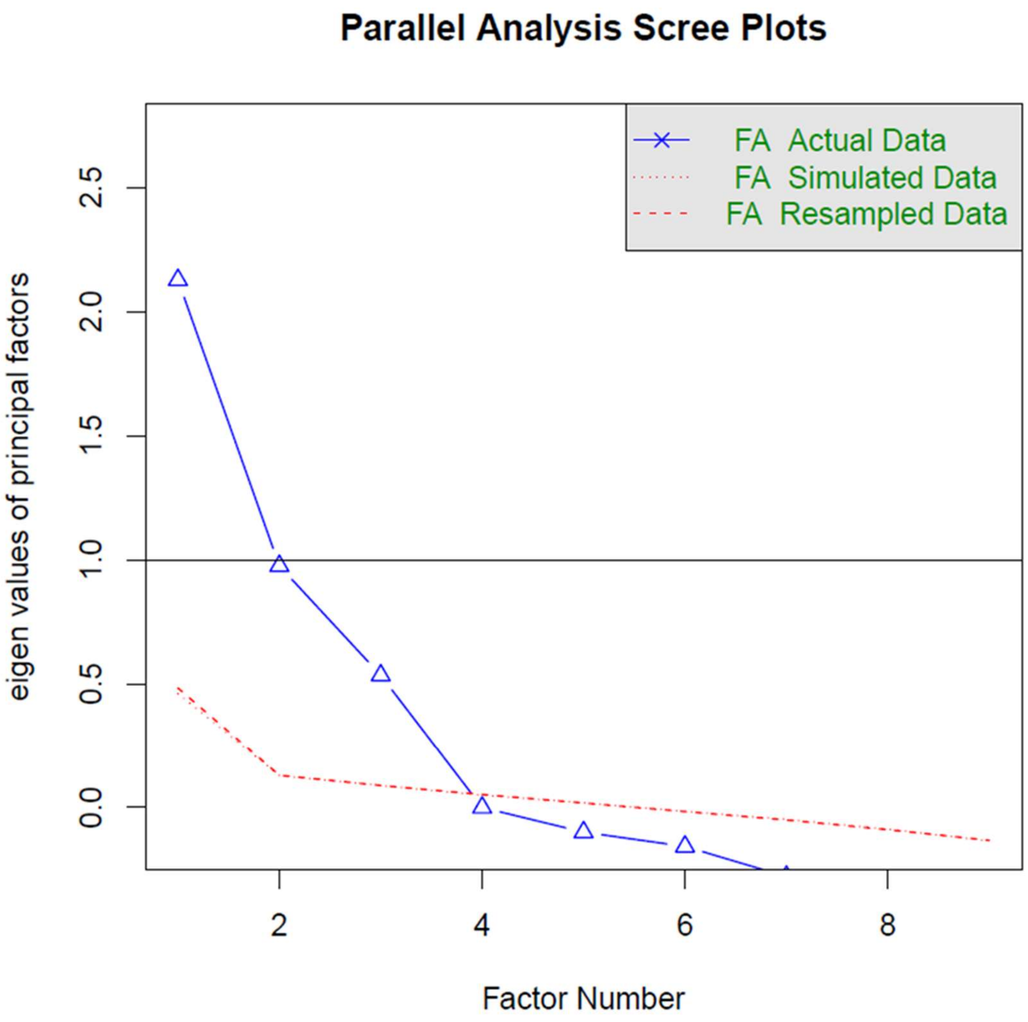

Supplementary Figure S2. Parallel analysis scree plot to determine the number of factors for factor analysis.

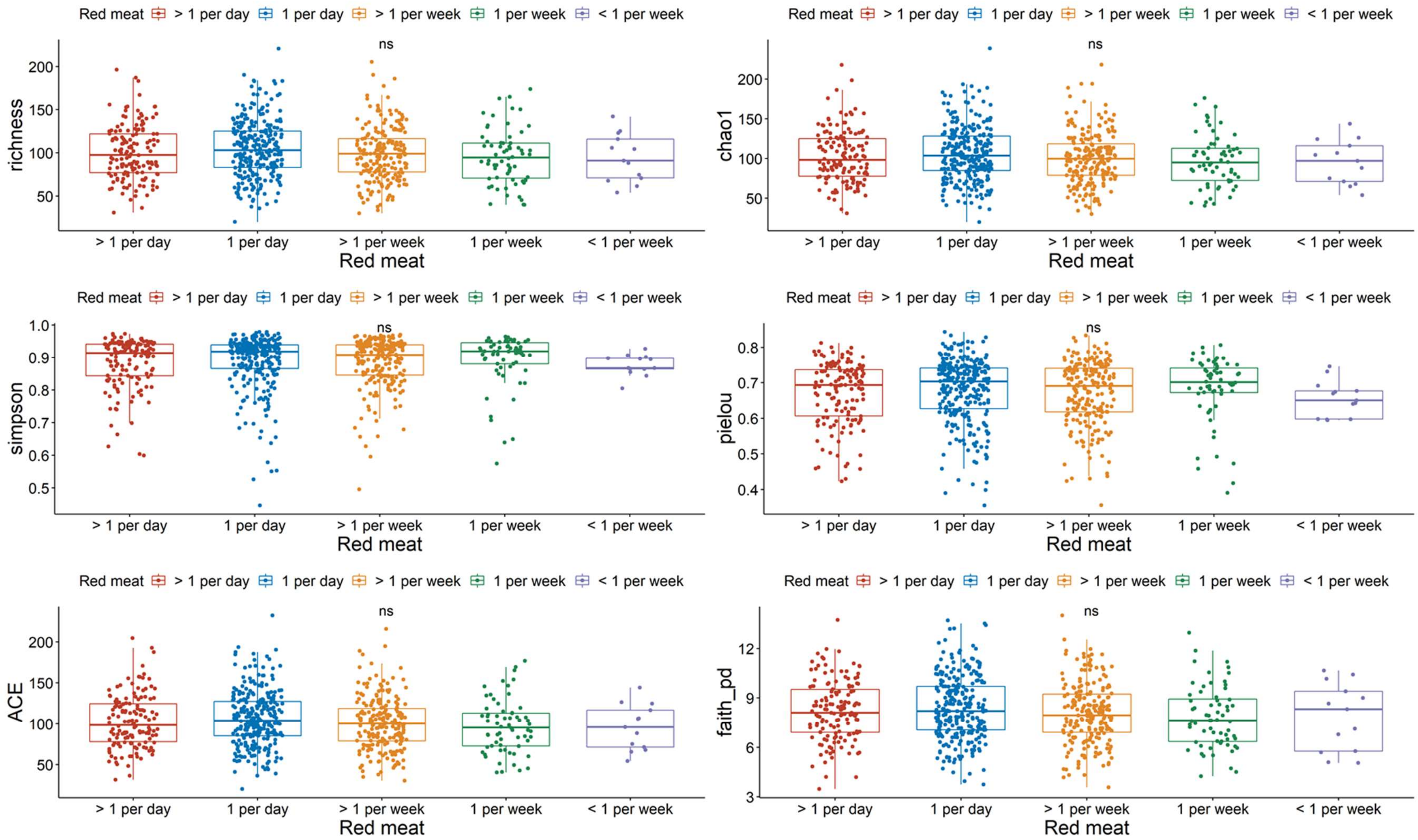

Supplementary Figure S3. α-diversity indexes (richness, chao 1, simpson index, pielou index, ACE and faith-pd index in different intake frequency groups of red meat.

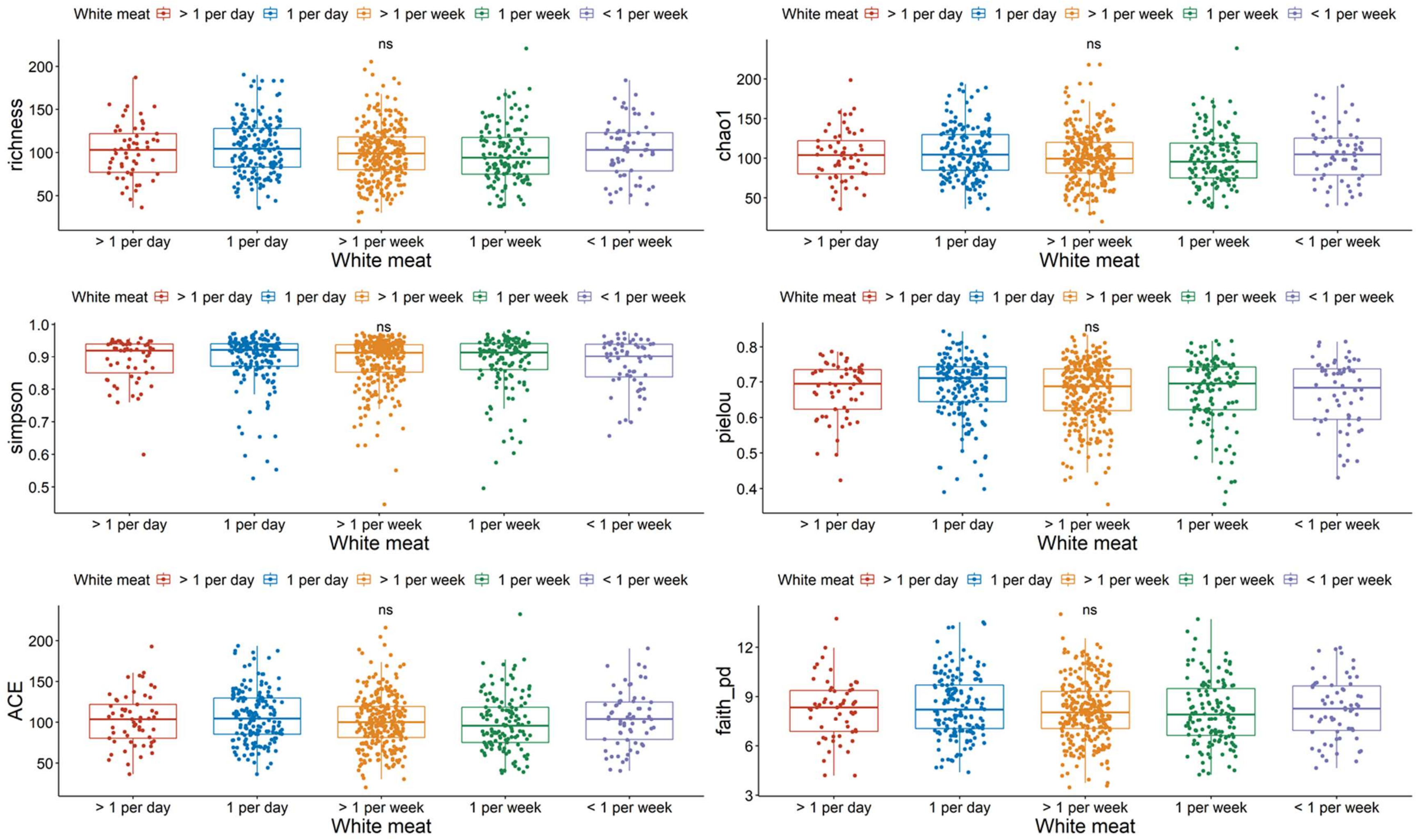

Supplementary Figure S4. α-diversity indexes (richness, chao 1, simpson index, pielou index, ACE and faith-pd index in different intake frequency groups of white meat.

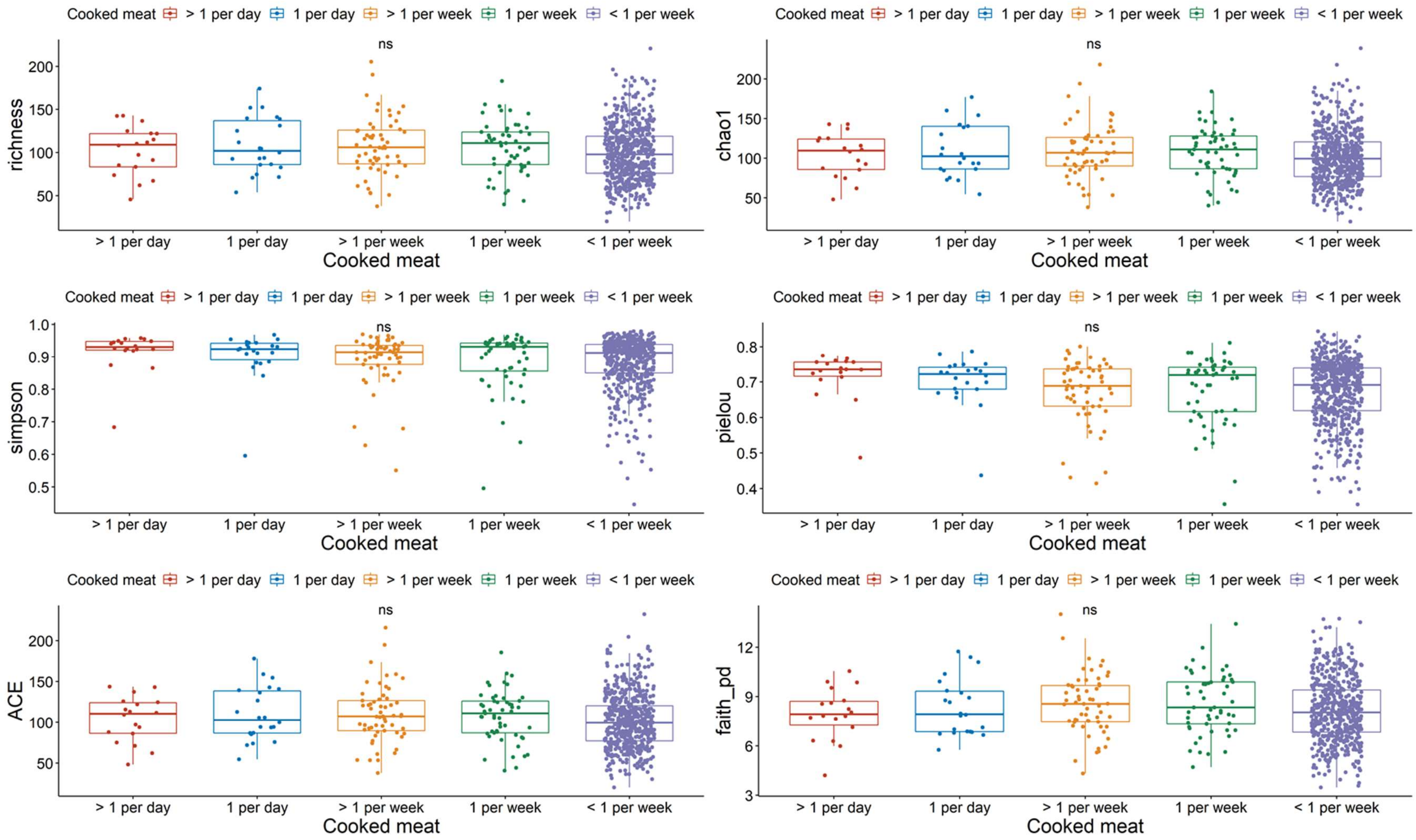

Supplementary Figure S5. α-diversity indexes (richness, chao 1, simpson index, pielou index, ACE and faith-pd index in different intake frequency groups of cooked meat.

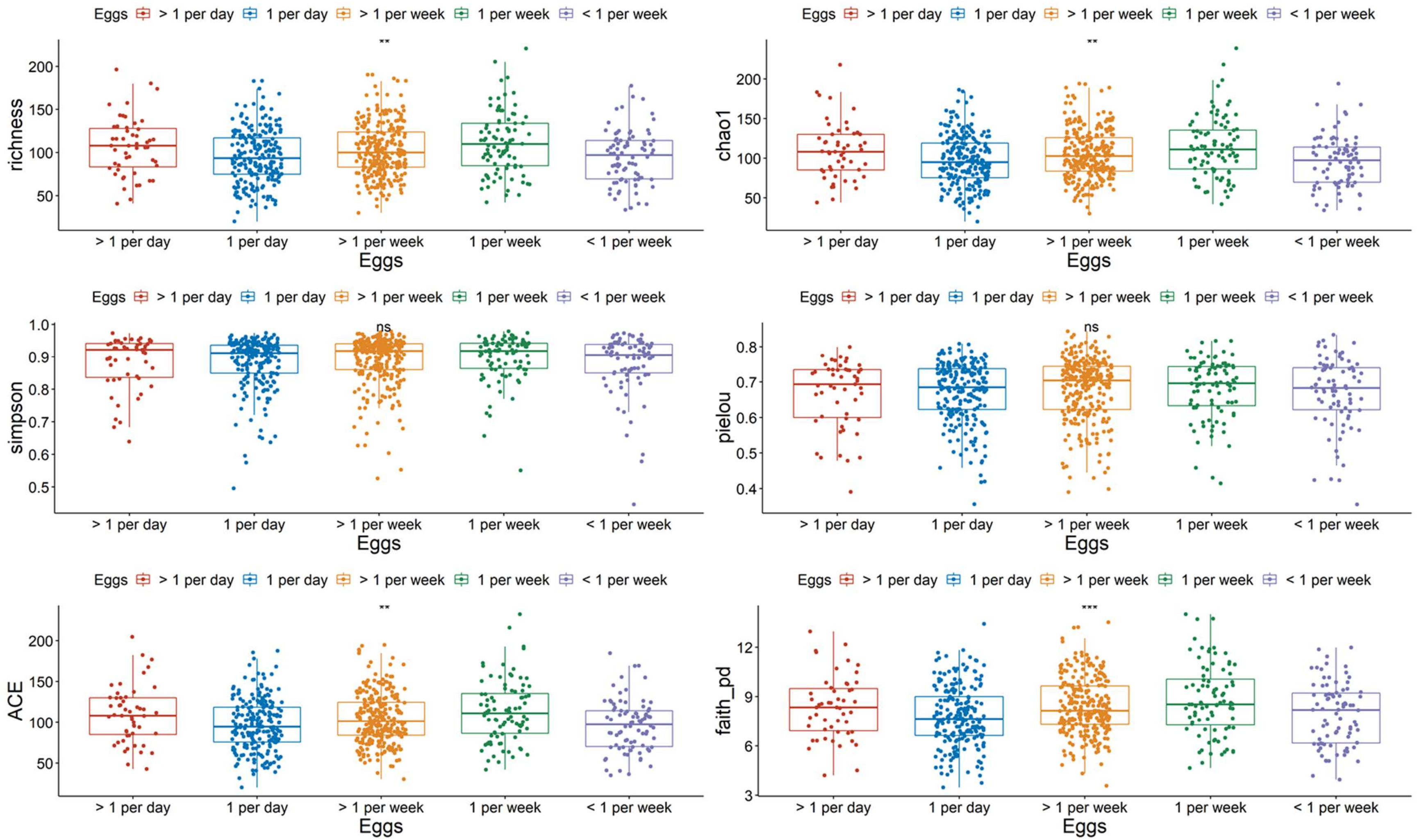

Supplementary Figure S6. α-diversity indexes (richness, chao 1, simpson index, pielou index, ACE and faith-pd index in different intake frequency groups of eggs.

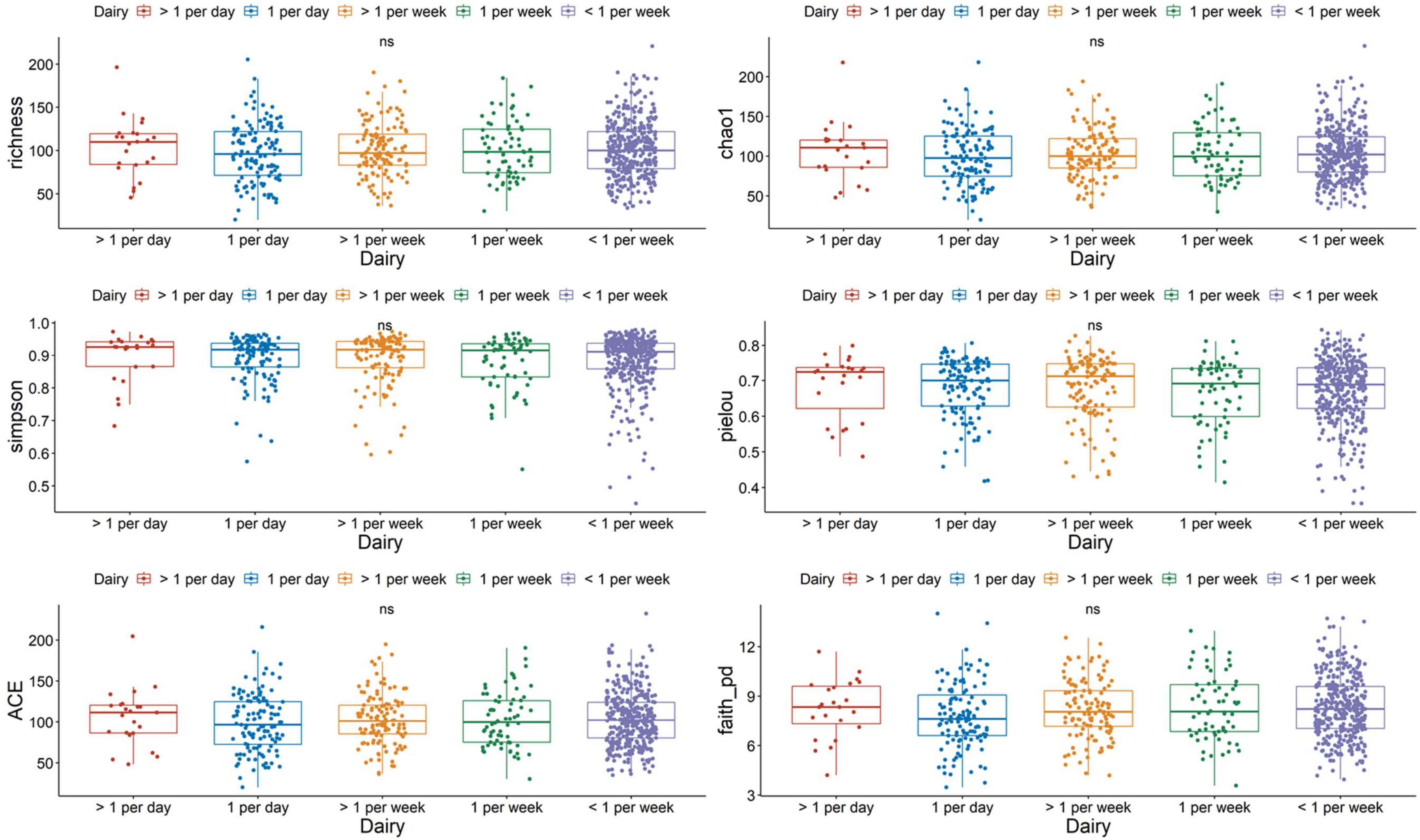

Supplementary Figure S7. α-diversity indexes (richness, chao 1, simpson index, pielou index, ACE and faith-pd index in different intake frequency groups of dairy products.

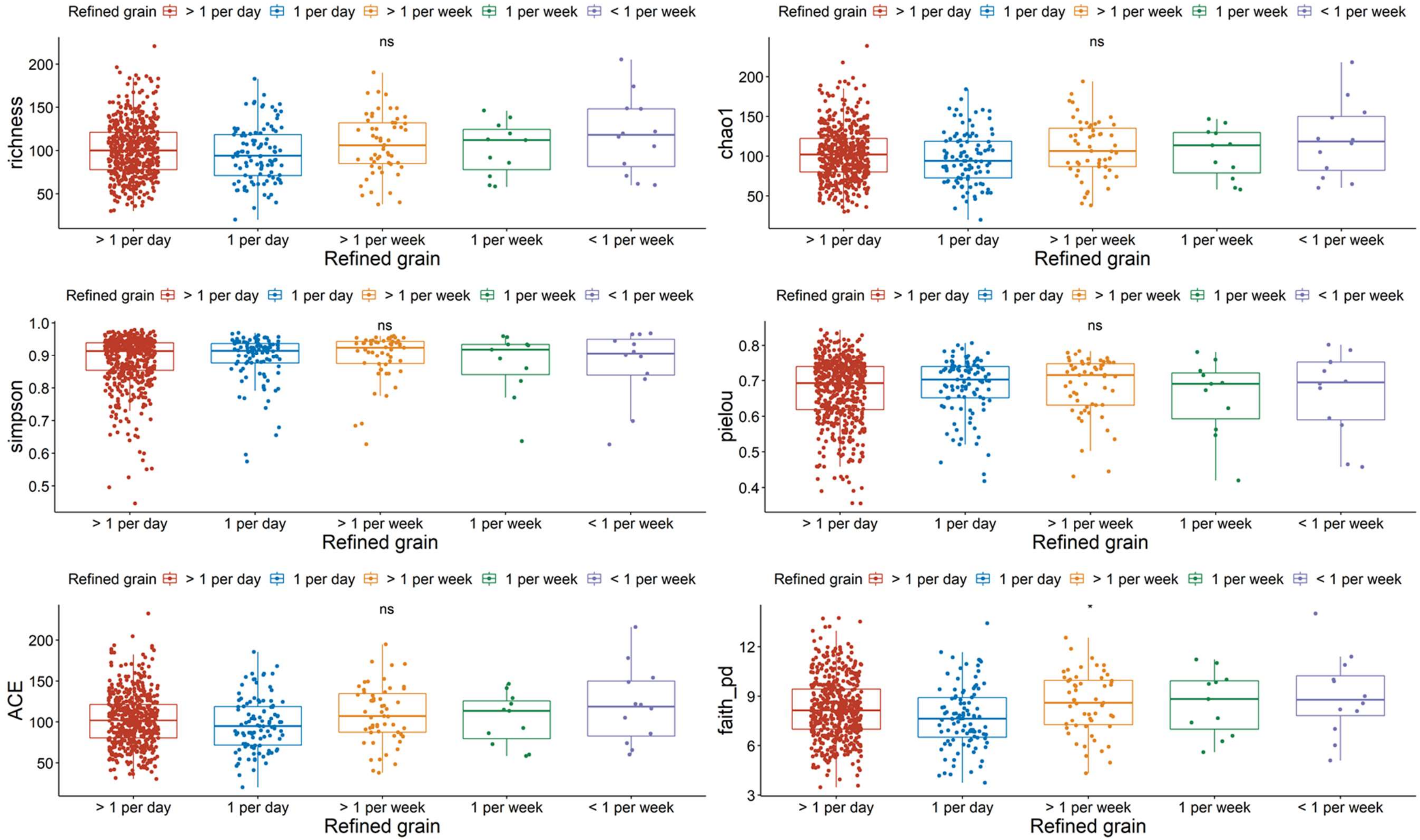

Supplementary Figure S8. α-diversity indexes (richness, chao 1, simpson index, pielou index, ACE and faith-pd index in different intake frequency groups of refined grain.

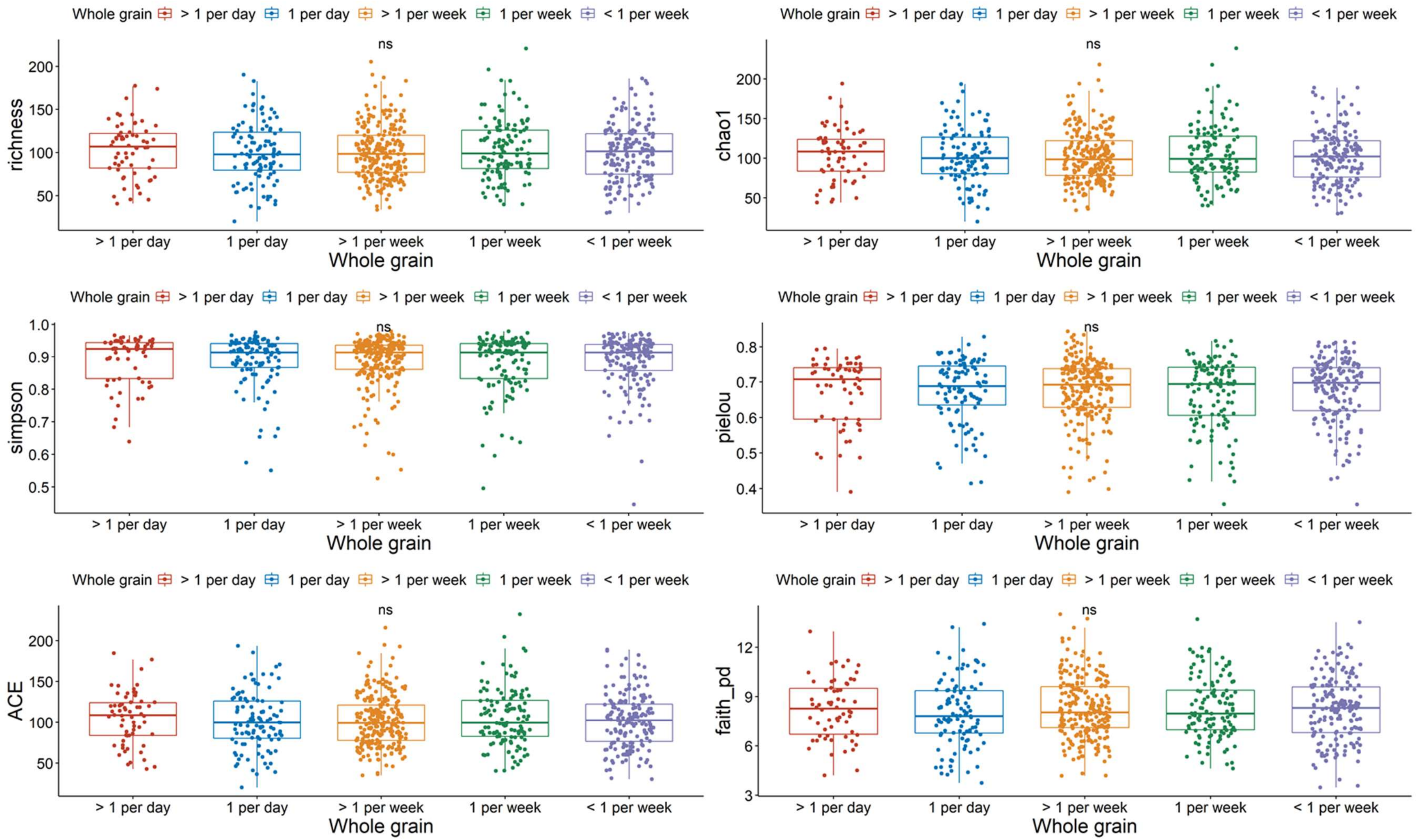

Supplementary Figure S9. α-diversity indexes (richness, chao 1, simpson index, pielou index, ACE and faith-pd index in different intake frequency groups of whole grain.

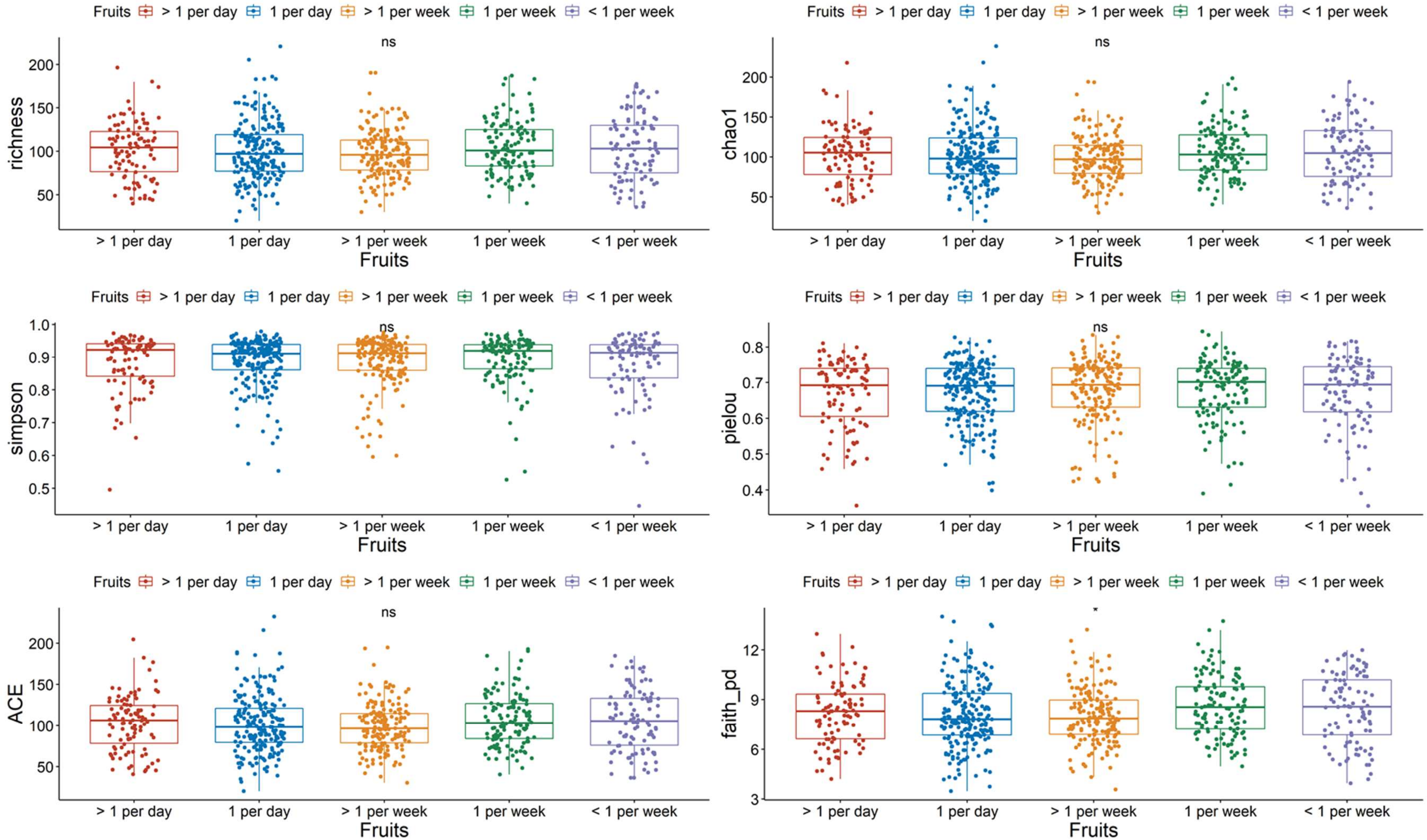

Supplementary Figure S10. α-diversity indexes (richness, chao 1, simpson index, pielou index, ACE and faith-pd index in different intake frequency groups of fruits.

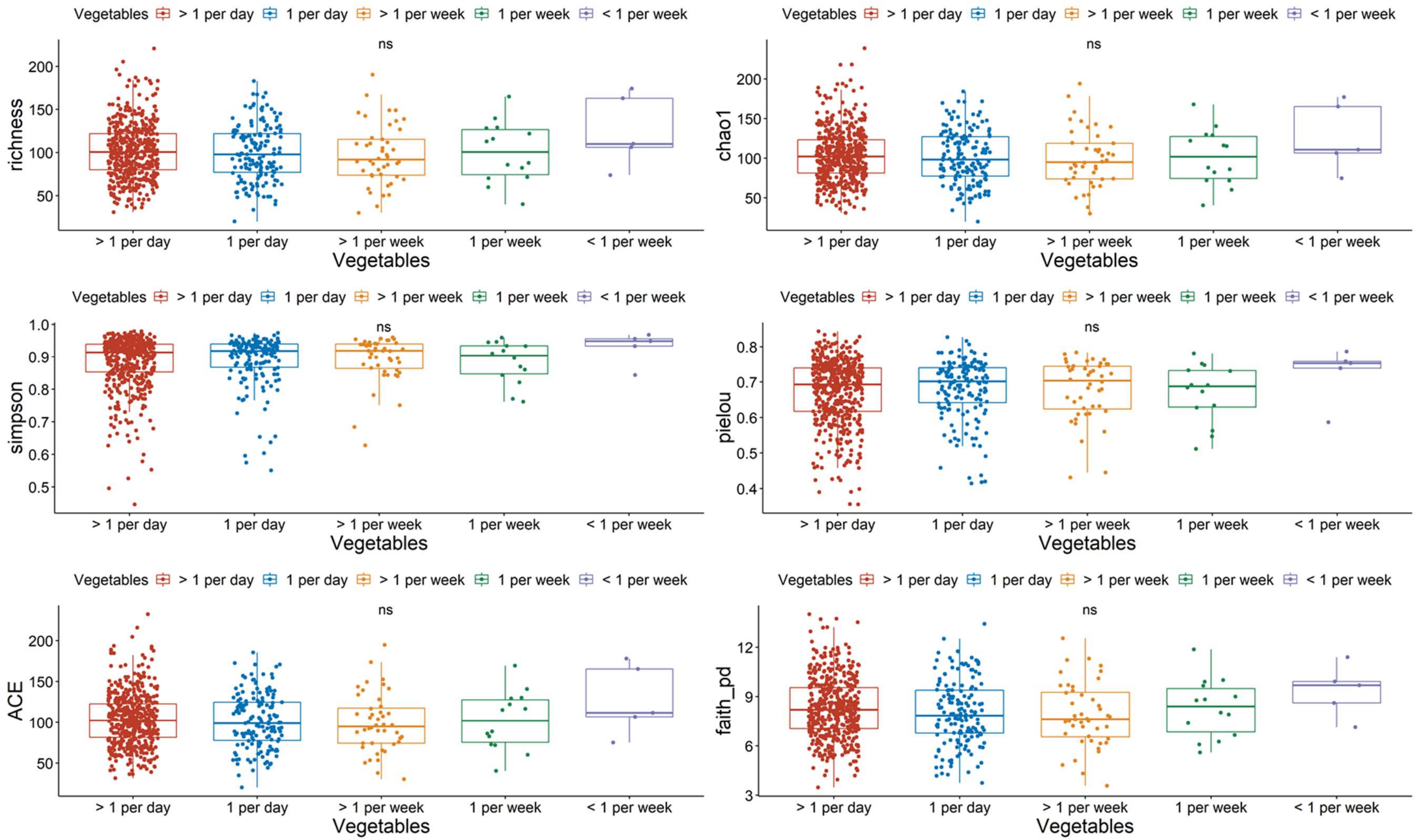

Supplementary Figure S11. α-diversity indexes (richness, chao 1, simpson index, pielou index, ACE and faith-pd index in different intake frequency groups of vegetables.
